# Supplementary material for: Genotype imputation in F2 crosses of inbred lines
Source: Bioinform Adv. 2024 Jul 23;4(1):vbae107. doi: 10.1093/bioadv/vbae107 (PMC11286293; doi:10.1093/bioadv/vbae107)
Supplement: vbae107_Supplementary_Data [file vbae107_supplementary_data.pdf]

Supplementary notes

Ideal sequencing depth and sample size in different populations

Our results indicate that a minimum sequencing depth of 0.5x is required for 2177 samples from our complex F2 cross setup in medaka fish. In Davies *et al.*, 2016 a similar sequencing depth versus sample size analysis is performed on 2073 CFW mice (Nicod *et al.*, 2016) (outbred population established from 2 founders) and on 11670 Han Chinese women from the CONVERGE study (Cai *et al.*, 2015). For the CFW mice, the first noticeable drop in imputation performance in the full sample set can be observed between 0.06x and 0.09x. For the CONVERGE study, the sample size does not seem to have a large impact on performance. For a sample size of 2000, a noticeable difference in correlation can be observed when moving from a sequencing depth of 1.4x to a sequencing depth of 1x.

This showcases how a more diverse population (CONVERGE study) requires higher sequencing depth for good imputation, while a less diverse population (CFW mice) can be reliably imputed with a lower sequencing depth. From our observations in terms of required sequencing depth, our medaka population is somewhat intermediate between these 2 examples.

Use of the info score for filtering variants

We tested whether the info score provided by STITCH can be a suitable replacement for the external validation approach based on high-coverage sequencing that we used. We observe that the rank-based Spearman correlation between the info score and the external validation r^2 is 0.58 for the full SNP set prior to filtering, and 0.51 for the refined SNP set after iterative filtering. These values were calculated excluding sites for which the validation r^2 was missing (for example, sites that are not polymorphic in the ground truth samples). The total number of retained sites was 6.2 million for the unfiltered set and 3.1 million for the filtered set. The relatively low correlation between the info score and the external validation performance indicates that external validation is to be preferred whenever possible.

References

Cai,N. *et al.* (2015) Sparse whole-genome sequencing identifies two loci for major depressive disorder. *Nature*, **523**, 588–591.

Davies,R.W. *et al.* (2016) Rapid genotype imputation from sequence without reference panels. *Nature Genetics*, **48**, 965–969.

Nicod,J. *et al.* (2016) Genome-wide association of multiple complex traits in outbred mice by ultra-low-coverage sequencing. *Nat Genet*, **48**, 912–918.

Supplementary figures

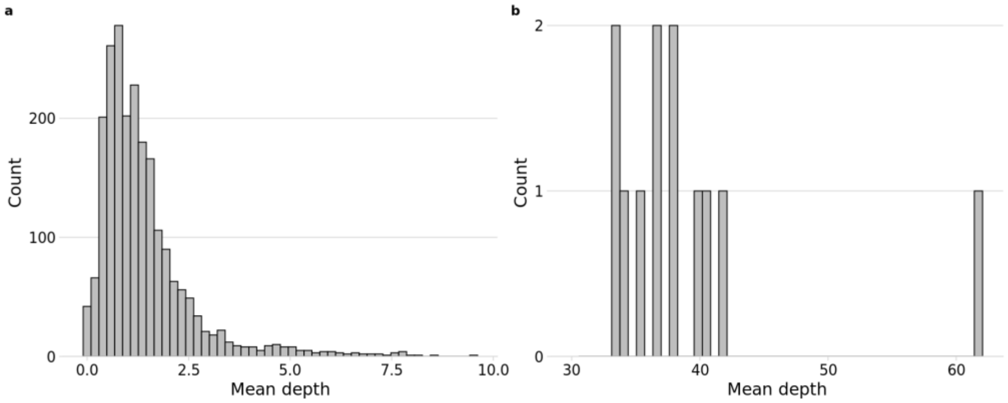

Figure S1. Distribution of mean sequencing depths in the original dataset used in this study. (a) Low-coverage samples. (b) Ground truth samples.

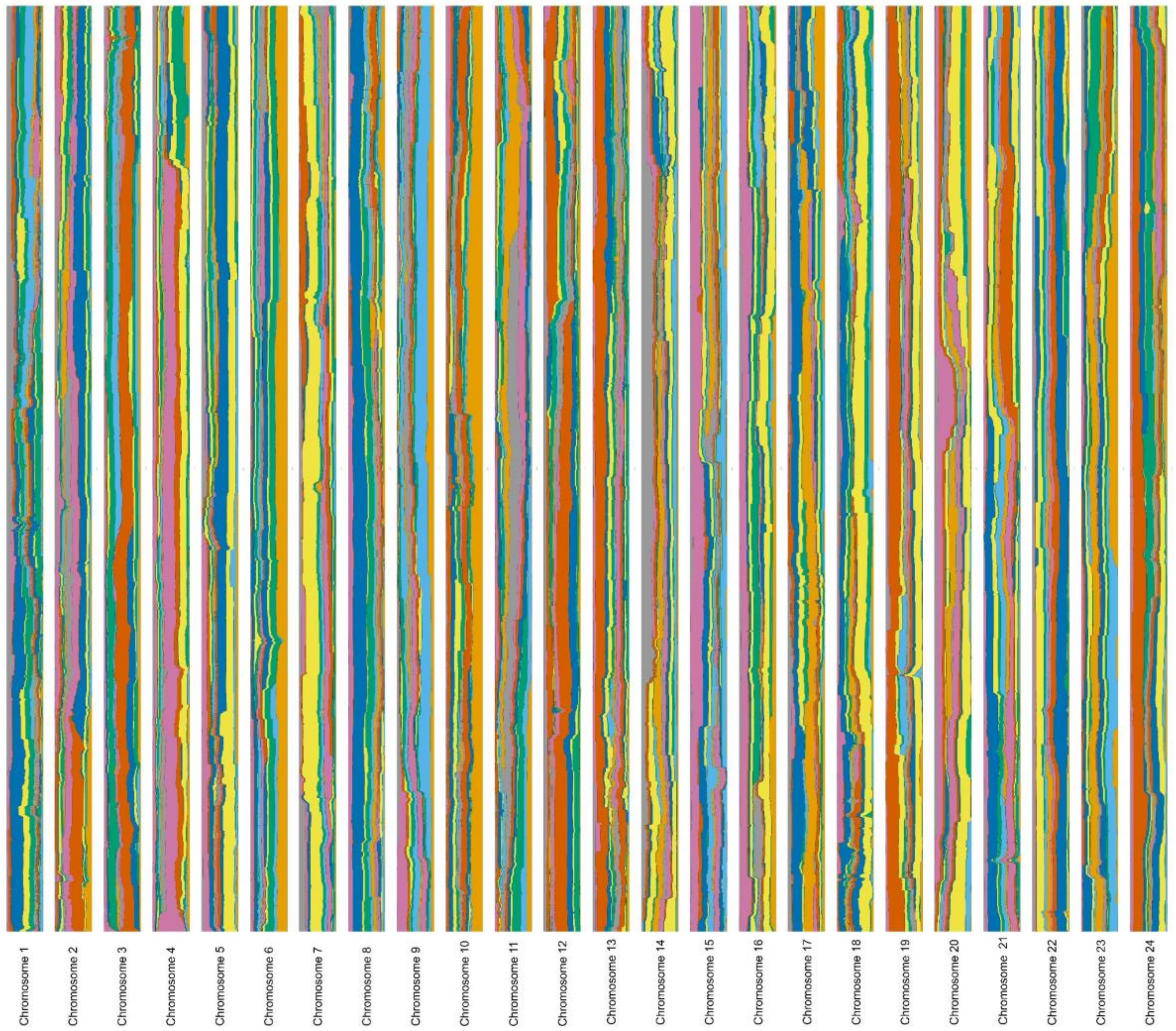

Figure S2. STITCH haplotype usage along the different chromosomes for the imputation run with the truth set samples downsampled to 0.5x mean depth and all the other samples left at the original depth. This visualisation has been produced by joining the per-chromosome plots automatically produced by STITCH. Long segments of contiguous colour reflect a lower number of ancestral haplotype switches, and hence a better imputation. Approximately equal usage of all the haplotypes also reflects more optimised heuristics.

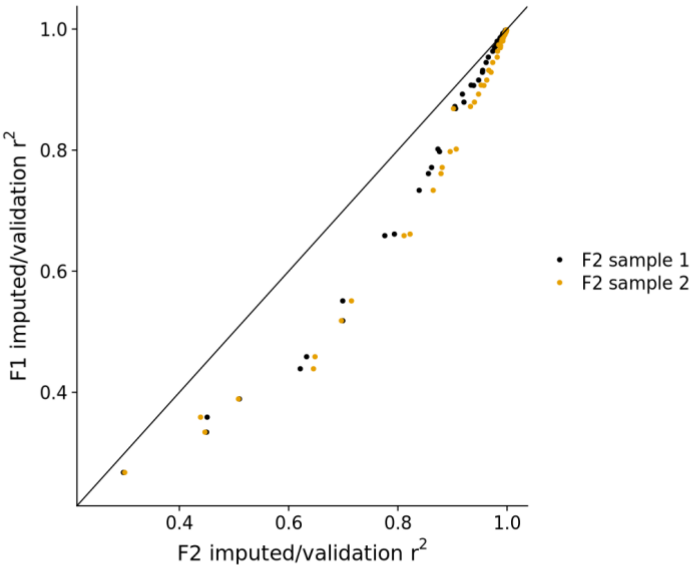

Figure S3. Comparison of the imputation performance evaluation on F1 samples and F2 samples from the same cross. For the F2 cross 72-2 x 55-2, we sequenced at high coverage one F1 fish and two F2 fish. This plot compares the sample-wise performance metrics for all the imputation runs performed in this work (each point is an imputation run) evaluated on the F1 sample (y-axis) and on the F2 samples (x-axis, different colours represent different F2 samples). F1 sample performance is an underestimation of the performance on F2 samples at intermediate correlation levels, while it tends to agree with the F2 performance towards the boundaries (0, 1). Importantly, the ranks of different performance runs are preserved (Spearman rank correlation = 0.99 for both F2 samples against the F1 sample), and so the F1 performance can be safely used for model selection in place of F2 performance.

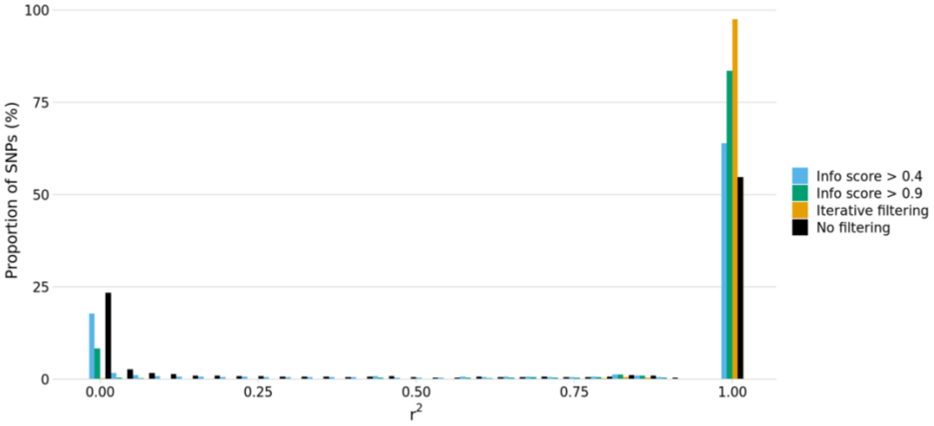

Figure S4. Distribution of SNP-wise imputation performance using different SNP filtering strategies. Iterative filtering is the approach that we used in this work, based on iterative imputation and filtering based on a comparison to the ground truth. The info score is an imputation quality metric internally produced by STITCH. Before filtering, many SNPs with very low imputed/validation r^2 are present, while after iterative filtering almost exclusively well-imputed SNPs are retained. On the contrary, filtering on the STITCH info score leads to inferior enrichment in well-imputed SNPs both at a 0.4 and at a 0.9 threshold. Note especially the large number of SNPs with very low ground truth correlation that are retained when filtering on the info score. The total number of SNPs is 6.42M for the original set, 5.39M for info score > 0.4, 3.2M for info score > 0.9, and 3.2M for the iterative filtering approach.

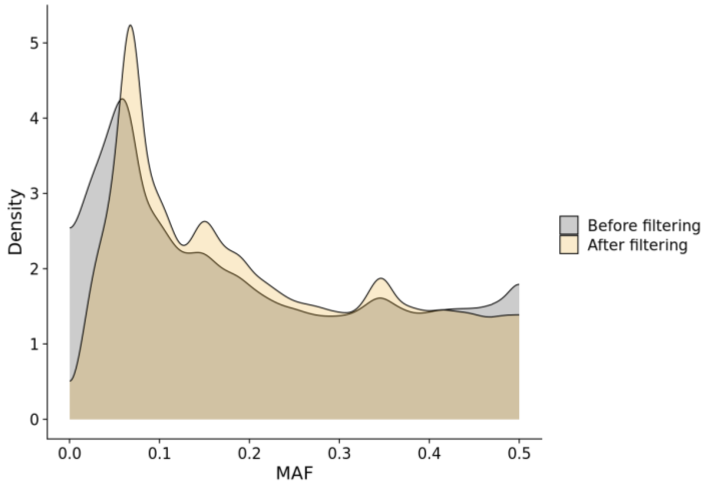

Figure S5. Distribution of the Minor Allele Frequency (MAF) of SNPs before and after the iterative filtering procedure described in this work. We observe that filtering preferentially retains SNPs with a MAF of approximately 0.06, while it depletes very rare or very common variants. A MAF of 0.06 is consistent with the frequency of a SNP being fixed in one founder line and absent from all the other founder lines when the line is used in 2 separate F2 crosses of 150 samples each (the most common scenario in our design). Non-variable SNPs (MAF = 0) were excluded.

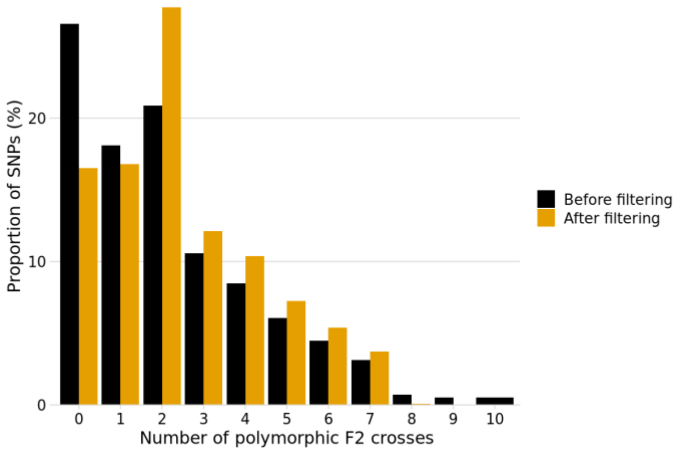

Figure S6. Strain distribution pattern for SNPs before and after the filtering procedure used in this work. We see an enrichment in SNPs segregating in 2 to 7 F2 crosses. Most founder lines are used in 2 separate F2 crosses in our design, while one founder line (72-2) is used in 7 crosses and one (22-1) is used in one cross only. We define a SNP as polymorphic in one cross when its minor allele frequency within the cross is greater than 0.4. Non-variable SNPs (MAF = 0 across all the samples) were excluded.
